# Tumor-suppressor role of miR-139-5p in endometrial cancer

**DOI:** 10.1186/s12935-018-0545-8

**Published:** 2018-04-02

**Authors:** JinHui Liu, ChunYu Li, Yi Jiang, YiCong Wan, ShuLin Zhou, WenJun Cheng

**Affiliations:** 1 Department of Gynecology, The First Affiliated Hospital of Nanjing Medical University, Nanjing, 210029 Jiangsu China; 2 Emergency Center, The First Affiliated Hospital of Nanjing Medical University, Nanjing, 210029 China

**Keywords:** miR-139-5p, HOXA10, Endometrial cancer, Viability, Migration

## Abstract

**Background:**

Endometrial cancer (EC) is the fourth most common malignancy of the female genital tract worldwide. MicroRNAs are important gene regulators with critical roles in diverse biological processes, including tumorigenesis. Several study’s show that miR-139-5p is involved in the tumorigenesis and metastasis of various cancers. However, its expression and potential biologic role in endometrial cancer remain to be determined. This study aimed to investigate the miR-139-5p expression and to analyze its function and underlying molecular mechanism in endometrial cancer.

**Methods:**

Expression of miR-139-5p was measured using qRT-PCR. The expression of HOXA10 was detected by Immunofluorescence staining in endometrial cancer tissues and adjacent normal tissues. CCK-8 and colony formation assays were used to assess the effect of miR-139-5p on ECC1 and Ishikawa cell line proliferation. Transwell migration assay was used to study the effect of miR-139-5p on EC cell migration. Luciferase reporter assay and western blot were used to confirm targeting of HOXA10 by miR-139-5p.

**Result:**

We demonstrated that miR-139-5p was down-regulated in human endometrial cancer compared to their matched adjacent non-tumor tissues. Overexpressed miR-139-5p significantly inhibited endometrial cancer cell viability and migration. Computational algorithm in combination with dual luciferase reporter assays identified HOXA10 as the target of miR-139-5p. HOXA10 expression was downregulated in endometrial cancer cells after miR-139-5p overexpression. The expression level of HOXA10 was significantly increased in endometrial cancer tissues, which was inversely correlated with miR-139-5p expression in clinical endometrial cancer tissues.

**Conclusion:**

These findings indicate that miR-139-5p targets the HOXA10 transcript and suppresses endometrial cancer cell growth and migration, suggesting that miR-139-5p acts as a tumor suppressive role in human endometrial cancer pathogenesis.

**Electronic supplementary material:**

The online version of this article (10.1186/s12935-018-0545-8) contains supplementary material, which is available to authorized users.

## Background

Endometrial cancer (EC) is the most frequent gynecological cancer and the fourth most common cancer for women in the developed world [1]. Despite more than 70% cases being diagnosed at the early stage, as much as 28% of patients have regional or distant metastasis. Unfortunately, their prognosis is usually poor, with a 5-year survival rate of < 40% [2]. The development of EC is a multistep process with accumulation of genetic and epigenetic alterations [3]. Most ECs are diagnosed in early clinical stages and can be managed surgically with good results. However, there is still a number of cases with bad prognosis due to lack of effective treatment for advanced, recurrent or disseminated disease. Approximately 80% ECs are of endometrioid histology [4]. So, it is critically important to develop novel therapeutic strategies for EC. Identifying the molecular mechanisms of EC carcinogenesis may help to understand of the pathogenesis of EC and therapeutic targets.

MiRNAs are small, non-coding RNA molecules that take part in RNA silencing and post-transcriptional regulation of gene expression. Altered expression of miRNAs may be associated with cancer initiation, progression and metastatic capabilities [5]. Many evidences have shown that miRNAs are involved in many cellular processes, including development, apoptosis, differentiation, metabolism and stress response, depending upon the regulation of specific target genes [6]. Some miRNAs promote the process of tumourigenesis and function as oncogenes in EC. For example, miR-10b inhibits apoptosis and promotes proliferation and invasion of EC cells via targeting HOXB3 [7]. MiR-135b can target FOXO1 to promote cell proliferation in EC cells [8]. Whereas, other miRNAs are expressed at a low expression level and act as tumor-suppressors. MiR-548c reduces the migration and invasion of endometrial and ovarian cancer cells via downregulation of Twist [9]. MiR-126 inhibits the migration and invasion of EC cells by targeting insulin receptor substrate 1 [10]. MiR-23a suppresses epithelial-to-mesenchymal transition in endometrial endometrioid adenocarcinoma by targeting SMAD3 [11]. Recently many miRNAs are closely associated with malignancy, including facts such as tumor progression, potential prognostic markers and chemotherapy resistance in endometrial cancers [5, 12, 13]. In the tumor-associated miRNAs, the mechanism of miR-139-5p in cancer initiation and progression drew our attention, because miR-139-5p has been found to perform various biological functions in other types of malignancy, such as bladder, colon, esophageal, liver and lung cancer [14–17]. However, there has not been any thorough research investigating the association between miR-139-5p and EC.

HOXA10 is a member of the *HOX* gene family that regulates embryonic morphogenesis [18]. Its aberrant expression was first observed in leukemia [19]. Both up-regulation and down-regulation of HOXA10 expression have been associated with cellular processes implicated in cancer, including proliferation, apoptosis, epithelial–mesenchymal transition (EMT) and treatment resistance, which include the relation between HOXA10 and EC [15, 20–22]. Previously, we showed that HOXA10 regulates G1 phase arrest in endometrial cancer which may be mediated by p21 [22]. However, the regulatory mechanisms that underlie the expression of HOXA10 in EC have yet to be studied. We predicted that miR-139-5p, is a regulator of HOXA10 expression, based on PicTar, TargetScan, and miRBase database. However, the role of miR-139-5p in EC development, especially regarding its link with HOXA10, has not been explored yet. In the present study, we investigated the expression of miR-139-5p in human EC and paired adjacent normal tissues, and explored the effects of miR-139-5p on cell growth and migration in vitro. Our results suggest that miR-139-5p has as a tumor suppressive role in human EC pathogenesis.

## Methods

### Tissue samples and cell lines

Informed consent for the analysis of tissue specimens in this study was obtained from every patient. This research was approved by the Institutional Review Board of Nanjing Medical University. EC patient tissues were immediately frozen and stored at − 80 °C after resection until use. 25 endometrial cancer tissues and 15 of normal tissues were excised surgically from patients with informed consent in the Department of Gynecology and Obstetrics, the First Affiliated Hospital of Nanjing Medical University, during period from 2014 and 2015, 11 of them are paired. Endometrial carcinoma cell lines (ECC1, Ishikawa) were grown in RPMI-1640 medium with 10% fetal bovine serum (FBS) at 37 °C, 5% CO_2_.

### Quantitative real-time RT-PCR (qRT-PCR) analysis

Quantitative real-time RT-PCR (qRT-PCR) was carried out using the protocol provided by the manufacturer. Total RNA was extracted from endometrial carcinoma tissues using TRizol reagent (Thermo Fisher Scientific, Waltham, MA, USA) according to the manufacturer’s protocol. The integrity of isolated RNA was evaluated by the Agilent Bioanalyzer 2100 with RNA 6000 Nano kit (Agilent Technologies, Santa Clara, CA, USA). Single-stranded complementary DNA was synthesized from 1 μg RNA in a 20 μL reaction volume using the high-capacity cDNA reverse transcription kits (Thermo Fisher Scientific), and the reaction was performed according to the manufacturer’s protocol. Real-time quantification of miRNA was performed using a SYBR Green PCR kit (Thermo Fisher Scientific), and the cycle threshold (Ct) of each gene was recorded. The relative expression of miR-139-5p was normalized to U6, and calculated using the 2^−ΔΔCt^ method (ΔCt = Ct^target gene^ − Ct^internal control^) [23]. The real-time polymerase chain reaction (PCR) conditions were as follows: 95 °C 10 min; 40 cycles of 95 °C 15 s, 67 °C 30 s, 72 °C 30 s, and 72 °C 5 min. The primers used are shown in Additional file 1.

### Protein extraction and western blot analysis

The cells were harvested after 48 h of transfection, washed twice with phosphate-buffered saline, and lysed in radio immunoprecipitation assay (RIPA) lysis with 0.01% protease and phosphatase inhibitor, respectively, and incubated on ice for 30 min. Cell lysis was centrifuged at 14,000 rpm for 15 min, and the supernatant (50 μg) of total protein was run on 10% sodium dodecyl sulfate–polyacrylamide gel electrophoresis and transferred electrophoretically to a polyvinylidene fluoride membrane (EMD Millipore, Billerica, MA, USA). After blocking in western blocking reagent (10%; Hoffman-La Roche Ltd, Basel, Switzerland), the membrane was incubated overnight at 4 °C with polyclonal rabbit anti-human HOXA10 (1:000; Abcam, ab90641, USA) and mouse anti-human GAPDH (1:1000, Hoffman-La Roche Ltd), respectively, and then the blot was incubated with DyLight^®^-conjugated secondary antibody (Rockland, ME, USA) at room temperature for 1 h, then visualized and analyzed using the Odyssey IR imaging system (LI-COR Biosciences, Lincoln, NE, USA).

### miRNA transfection

miR-139-5p mimics were synthesized by GenePharma (Shanghai, People’s Republic of China). A total of 5 × 10^5^ endometrial carcinoma cells were seeded in each well of six-well plates and cultured in serum-free RPMI-1640 medium for 1 day before transfection. The cells were transfected with 50 nM miR-135-5p mimics by using Lipofectamine^®^ 2000 reagent (Thermo Fisher Scientific), following the manufacturer’s protocol.

### Transwell migration assay

The migration assays of ECC1 and Ishikawa cells were conducted using Transwell chambers (Millipore, Billerica, MA) placed into a 24-well plate. For the migration assay, 1 × 105 cells were resuspended in 200 μL serum-free medium and placed in the top chambers. RPMI-1640 medium (600 μL) containing 10% FBS was filled into the bottom chambers. Cells were incubated for another 20 h at 37 °C with 5% CO_2_. After the incubation, the cells were fixed with polyoxymethylene. The numbers of migrated cells were calculated from five randomly fields.

### Colony formation assay

Colony formation assay was carried out using the protocol described previously and briefly [24], cells were transfected with NC, miR-139-5p, as described above. Twenty-four hours later, transfected cells were trypsinized, counted and replated at a density of 500 cells/6 cm dish. Ten days later, colonies resulting from the surviving cells were fixed with 3.7% methanol, stained with 0.1% crystal violet and counted. Colonies containing at least 50 cells were scored. Each assay was performed in triplicates.

### CCK-8 assay

Cell growth was measured using the cell proliferation reagent WST-8 (Roche Biochemicals, Mannheim, Germany). After plating cells in 96-well microtiter plates (Corning Costar, Corning, NY) at 1.0 × 10^3^/well, 10 μL of CCK8 was added to each well at the time of harvest, according to the manufacturer’s instructions. One hour after adding CCK8, cellular viability was determined by measuring the absorbance of the converted dye at 450 nm.

### Dual-luciferase reporter assay

The 3ʹ-untranslated regions (3ʹ-UTRs) of human HOXA10 cDNA with the putative target sites for miR-139-5p (sequences were provided in Additional file 1) were synthesized and then inserted at the *Xba*I site downstream of the luciferase gene in the pGL3-control (Promega) vector by Integrated Biotech Solutions Co., Ltd (Shanghai, China).

Twenty-four hours before transfection, cells were seeded at 1.5 × 10^5^ cells per well in 24-well plates. Then, two hundred ng of pGL3-HOXA10-3ʹ-UTR plus eighty ng of pRL-TK (Promega) were co-transfected with 60 pmol of miR-139-5p mimic or mimic control using Lipofectamine 2000 (Invitrogen) following the manufacturer’s protocol. Luciferase activity was measured 24 h after the transfection using the Dual luciferase assay system (Promega), as previously described. The firefly luciferase activity was normalized to that of the Renilla luciferase for each well. Three independent experiments were performed in duplicate.

### Immunohistochemistry

Fifteen tissue samples were formalin-fixed and paraffin embedded cut to 4-μm thick, and stained using the avidin–biotin complex method. Tissue slides were subjected to antigen retrieval using microwave irradiation in 10 mol/L citrate buffer (pH 6.0), followed by incubation with primary antibodies at 4 °C overnight. The polyclonal antibody for HOXA10 was purchased from Abcam, Cambridge, MA, USA. Staining was repeated if the result was uncertain. The slides were scored independently by two observers blinded to clinicopathological characteristics. They evaluated the immunostaining of the slides under an optical microscope at a magnification of ×400. Discordant scores were reevaluated to reach consensus. The number of HOXA10 expression in positive cancer cells were assessed.

### Statistical analysis

All experiments were repeated 3 times independently. The results are presented as the mean ± standard error mean (SEM). Two independent sample *t*-test or one-way analysis of variance (ANOVA) was performed using SPSS 19.0 software in order to detect significant differences in measured variables among groups. A value of *P* < 0.05 was considered to indicate a statistically significant difference.

## Results

### Downregulation of miR-139-5p expression occurs in EC tissues

**Fig. 1:**
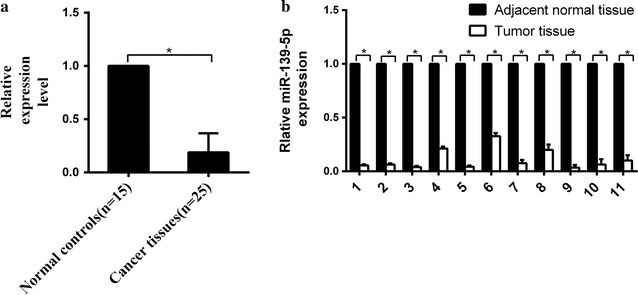
MiR-139-5p shows a low expression level in EC tissues. **a** The expression level of miR-139-5p in EC tissues (n = 25) and adjacent normal tissues (n = 15). Significantly lower expression of miR-139-5p was detected in EC tissues than that in adjacent normal tissues. *P < 0.05. **b** The quantitative analysis of miR-139-5p expression in 11 pairs of EC tissues and adjacent normal tissues. *P < 0.05

To determine the role of miR-139-5p in endometrial cancer development, we examined the expression of miR-139-5p in twenty-five endometrial cancer samples and fifteen adjacent normal tissues by RT-PCR. MiR-139-5p expression was significantly down-regulated in EC tissues compared to that in normal tissues (Fig. 1a). Furthermore, miR-139-5p expression was significantly downregulated in endometrial cancer tissues as compared to matched normal tissues by RT-PCR (Fig. 1b).

### HOXA10 is a target gene of miR-139-5p

**Fig. 2:**
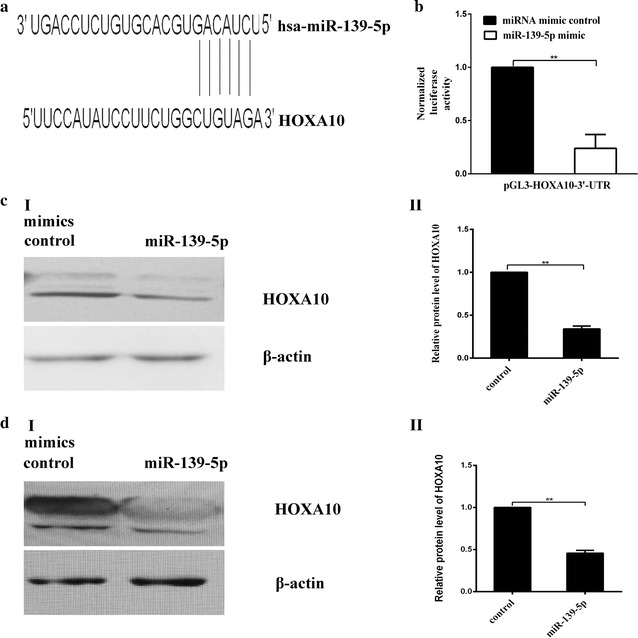
HOXA10 is a candidate target of miR-139-5p. **a** Computational analysis identified that HOXA10 may be a potential target of miR-139-5p, and the predicted binding sequences of HOXA10 3′-UTR and miR-139-5p were marked. **b** Dual luciferase assay performed in HEK-293T cells confirms that HOXA10 is the target gene of the miR-139-5p. A significant decrease in relative luciferase activity was noted when pGL3-HOXA10-3′-UTR was co-transfected with the miR-139-5p mimic but not with the miRNA mimic control. **c**, **d** I HOXA10 expression levels, by western blot analysis, in ECC1 and Ishikawa cells upon miR-139-5p or negative control reintroduction. **c**, **d** II Histograms represent densitometric analysis. All experiments were performed in triplicate. **P < 0.01

To explore the mechanisms of miR-139-5p regulation in EC, we searched for the potential targets of miR-139-5p using online software and database. Using these programs, we selected HOXA10 as a miR-139-5p target gene for further study, as HOXA10 was important for malignant behavior in other types of cancer (Fig. 2a). Next, we performed Western blotting to confirm downregulation of HOXA10 protein following transfection of miR-139-5p in Ishikawa and ECC1 EC cells. The protein expression levels of HOXA10 were significantly repressed in miR-139-5p-transfected cells in comparison with miR-control transfected cells (Fig. 2c, d). Furthermore, we explored whether HOXA10 is the target gene of the miR-139-5p. We constructed a luciferase reporter vector with the putative HOXA10 3′-UTR target site for the miR-139-5p downstream of the luciferase gene (pGL3-HOXA10-3′-UTR). Luciferase reporter vector together with the miR-139-5p mimic or the miRNA mimic control were transfected into HEK-293T cells. In HEK-293T cells, a significant decrease in relative luciferase activity was noted when pGL3-HOXA10-3′-UTR was co-transfected with the miR-139-5p mimic but not with the miRNA mimic control, suggesting that HOXA10 is the target gene of the miR-139-5p (Fig. 2b).

### MiR-139-5p is negatively associated with HOXA10

**Fig. 3:**
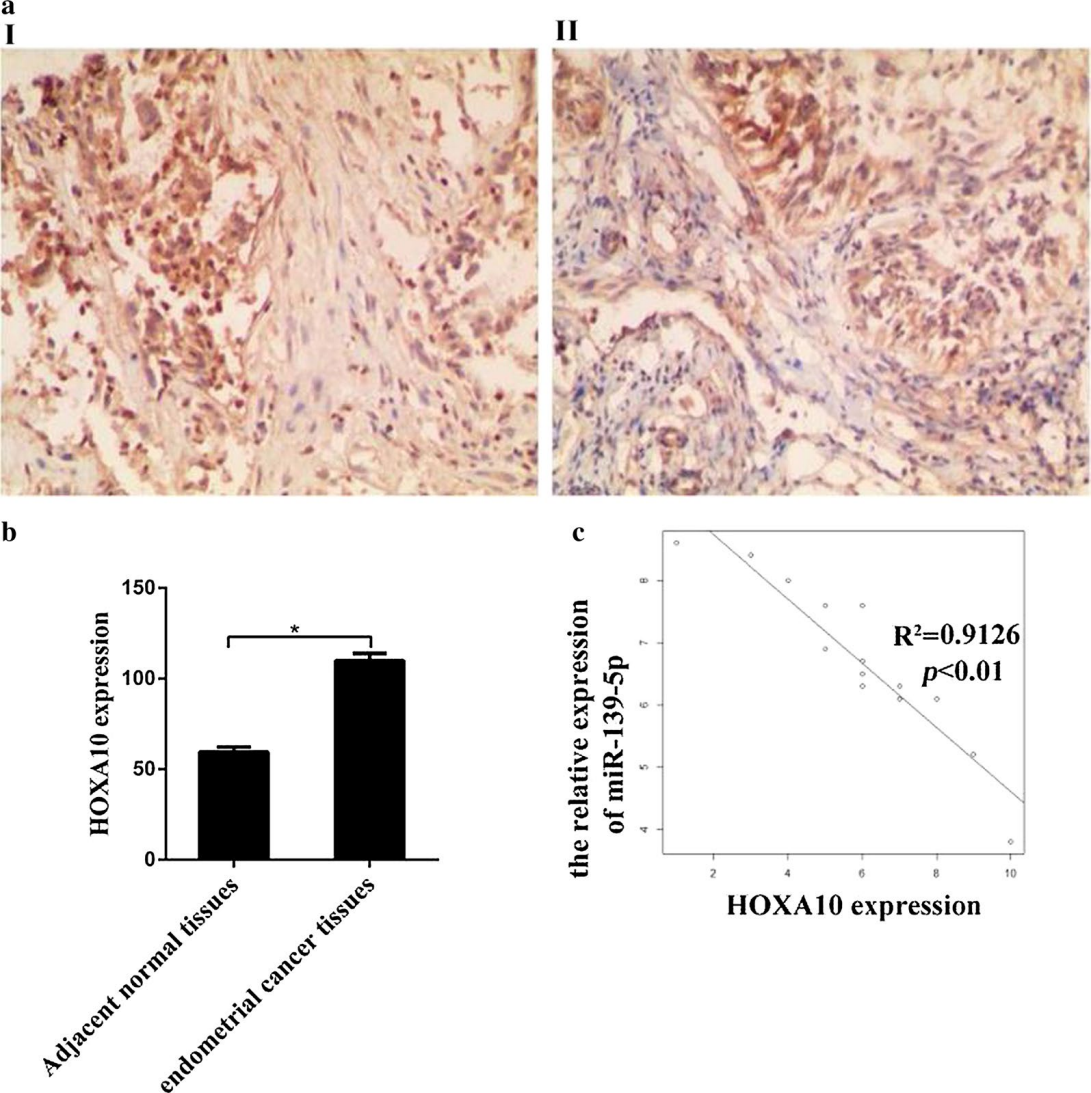
miR-139-5p is negatively associated with HOXA10. **a** Immunofluorescence staining showed that HOXA10 was significantly overexpressed in EC tissues, relative to adjacent normal tissues. Graphical presentation in **b**. **c** A plot of the relative expression of miR-139-5p vs HOXA10 showed an inverse correlation between the two items. The correlation index R^2^ was calculated using the Spearman’s rank test (R^2^ = 0.9126, P < 0.01). X axis was presented as IHC scores. Y axis was presented as relative expression (normalized to U6). *P < 0.05

To confirm the levels of HOXA10 expression in EC tissues, we utilized immunohistochemical staining to detect the HOXA10 protein levels. Fifteen EC and 8 normal endometrium tissues were subjected to HOXA10 protein expression analysis in this study. Immunohistochemical staining showed that the expression of HOXA10 protein was significantly higher in clinical EC specimens than that in adjacent normal tissues (Fig. 3a, b). An inverse correlation (R^2^ = 0.9126, P < 0.01) was observed between miR-139-5p and HOXA10 using Spearman’s correlation analysis (Fig. 3c).

### MiR-139-5p suppresses the viability and migration of the Ishikawa and ECC1 endometrial cancer cell lines

**Fig. 4:**
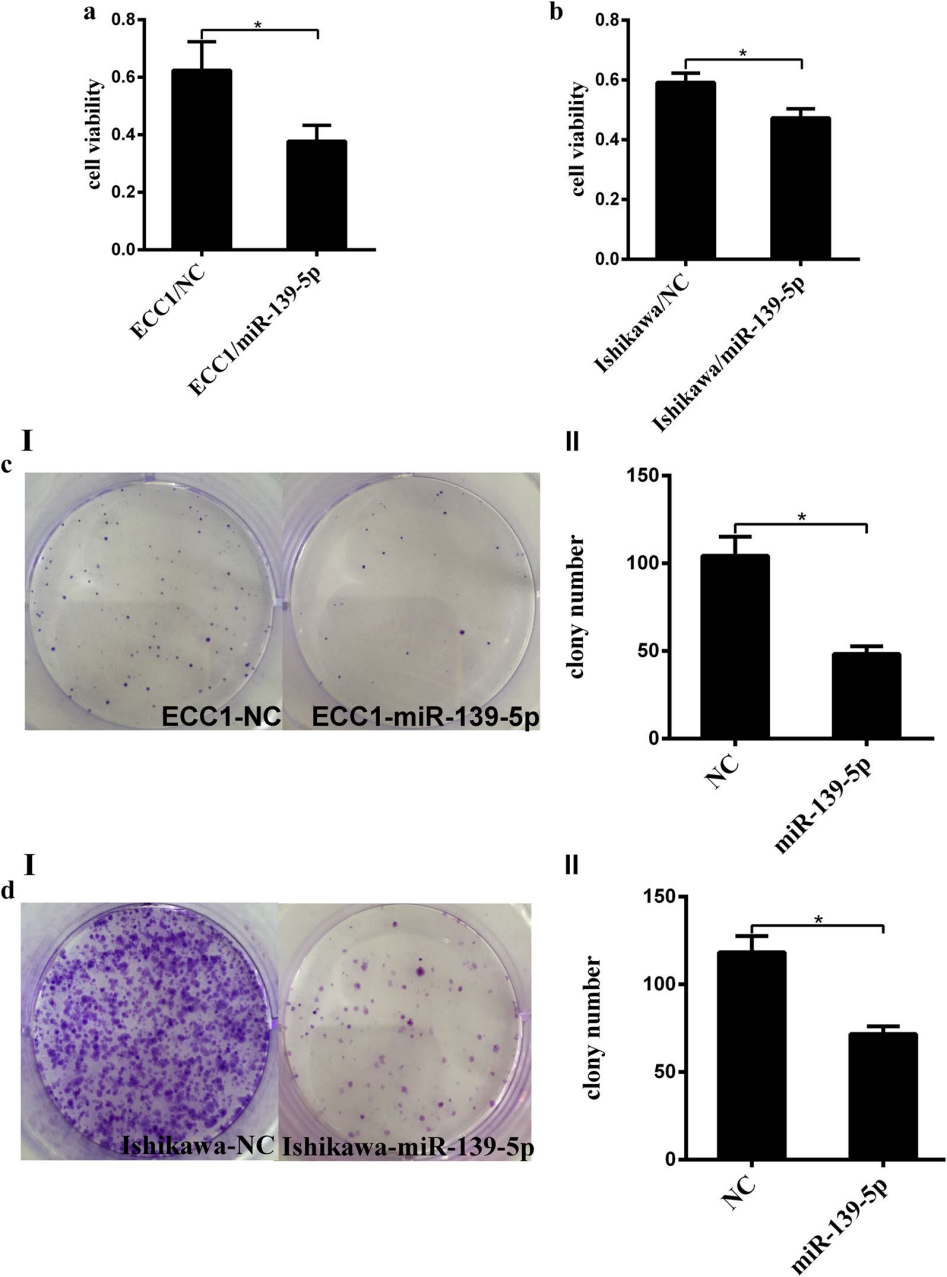
miR-139-5p significantly depresses the viability of EC cells. **a**, **b** Cell viability was detected using CCK-8 assay. After Ishikawa and ECC1 cells were transfected with the miR-139-5p mimics or mimics control, the CCK-8 assay was used to determine the relative cell growth activity at 1 h post-transfection. **c**, **d** Effect of miR-139-5p on cell viability as evaluated by a colony formation assay. Ishikawa and ECC1 cells transfected with miR-139-5p mimics or mimics control were seeded in 6-well plates. On the 10th day after seeding, the number of colonies was counted. *P < 0.05

**Fig. 5:**
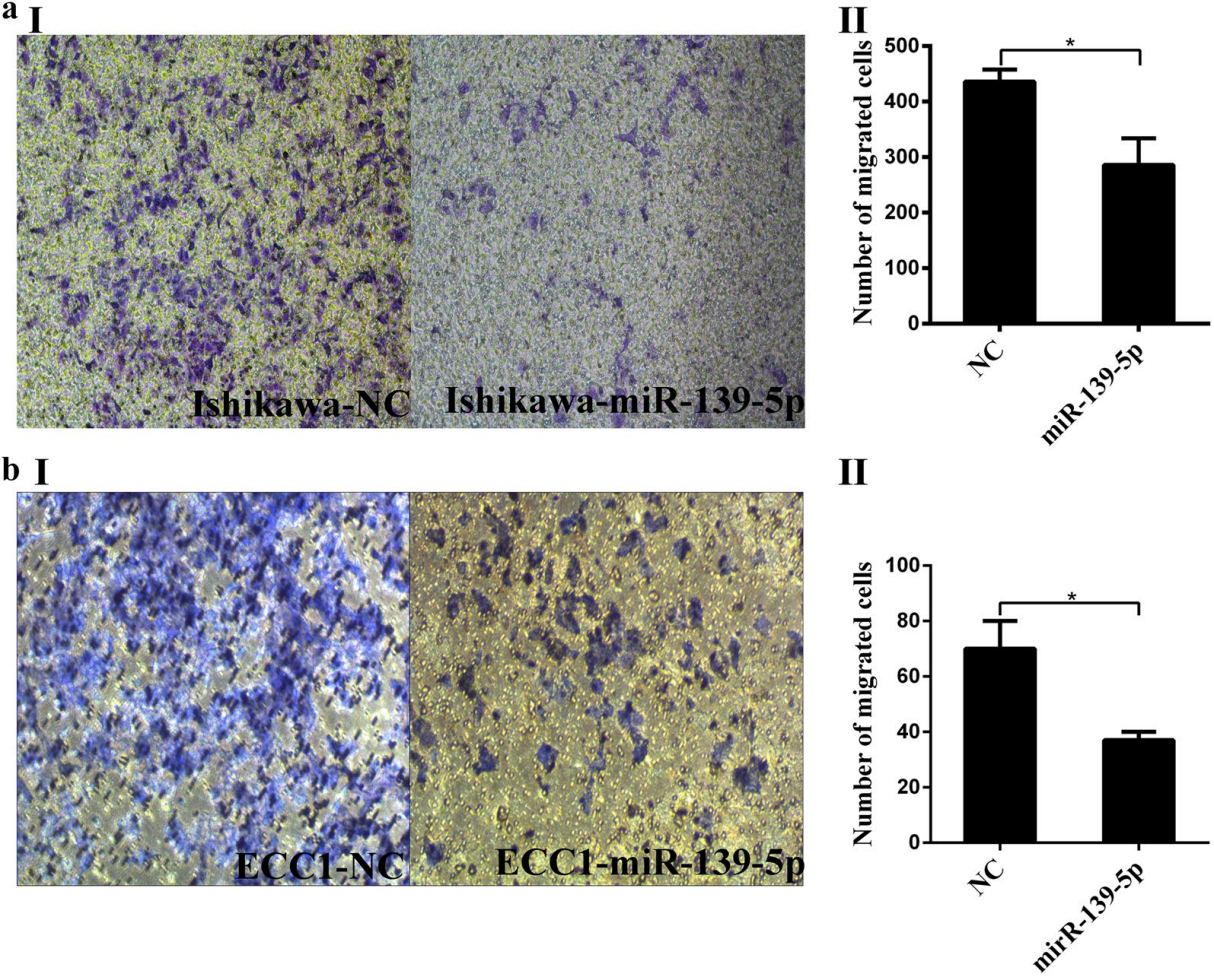
miR-139-5p depresses cell migration in EC cells. Ishikawa (**a**) and ECC1 cells (**b**) were transfected with miR-139-5p mimics or mimics NC for 48 h. The transwell chambers were employed for cell migration assays. The number of migrated cells of EC cells with miR-139-5p were decrease much more than the mimics NC group. *P < 0.05

The Cell viability and colony formation rate of Ishikawa and ECC1 cells transfected with miR-139-5p mimics, was significantly reduced as compared to that in the control group (Fig. 4a–d) miR-139-5p-overexpressed Ishikawa and ECC1 cells had the lower potential of migration when compared with the control group (Fig. 5).

## Discussion

Our findings are consistent with studies on miR-139-5p in other types of malignancy, such as bladder, colon, esophageal, liver and lung cancer [14–17, 25]. Yonemori et al. found that the expression of miR-139-5p/miR-139-3p in bladder cancer tissue is downregulated compared to normal bladder epithelia and overexpression of miR-139-5p/miR-139-3p in bladder cells significantly inhibited cell migration and invasion by targeting MMP11 [25]. Downregulation of miR-139-5p is also detected in esophageal squamous cell carcinoma (ESCC). Overexpression of miR-139-5p reduced ESCC cell proliferation, migration and invasion [14]. In their study on intestinal inflammation and colorectal cancer, Zou et al. found that miR-139-5p knockout mice are highly susceptible to colitis and colon cancer, accompanied by elevated proliferation and decreased apoptosis, as well as an increased production of inflammatory cytokines, chemokines and tumorigenic factors [17]. Similarly, overexpression of miR-139-5p also inhibits epithelial–mesenchymal transition, migration and invasion of hepatocellular carcinoma cells by targeting ZEB1 and ZEB2 [15]. In lung cancer, sun et al. showed that the level of miR-139-5p is significantly reduced in human lung cancer tissues and the low level of miR-139-5p correlates with survival of lung cancer patients [16]. Consistent with other types of cancer, transient introduction of miR-139-5p inhibiting cell proliferation, metastasis, and promoting apoptosis by targeting oncogenic c-Met in lung cancer cell line A549 and SK-MES-1 [16]. However, There are no any evidences investigating the association between miR-139-5p and EC. Here, we found that miR-139-5p acts as a tumor-suppressor gene involved in EC development.

HOX genes are members of the superfamily of homeobox genes encoding transcription factors, which is involved not only in cell proliferation, cell differentiation but also in tumorigenesis [26–28]. Deregulated expression of HOX genes has been observed in several cancer types, including glioblastoma, oral cancer, ovarian cancer and gastric cancers, etc. [21, 29–31]. HOXA10 is a member of the HOX gene family that regulates embryonic morphogenesis [18]. Recent studies have demonstrated that HOXA10 has been involved in regulation of proliferation, migration and invasion in oral squamous cell carcinoma [29]. Yang et al. reported that HOXA10 has been identified to be highly expressed in gastric cancer, and promotes gastric cancer cell proliferation, migration and invasion [30]. Jin et al. reported that HOXA10 was associated with the temozolomide resistance of glioblastoma cell [21]. Several HOX genes have been reported to be regulated partially by miRNAs [18]. For example, miRNA-135a has been reported to suppress cell proliferation, apoptosis, and adhesion by targeting HOXA10 in ovarian cancer [31]. In this study, target prediction software identified a miR-139-5p binding site in the 3′-UTR of HOXA10, and dual-luciferase reporter assays confirmed that HOXA10 is target of miR-139-5p. In confirmation of this functional interaction, overexpression of miR-139-5p reduced HOXA10 protein expression in EC cell lines.

## Conclusion

This study indicates the levels of miR-139-5p in EC tissues were markedly reduced compared to normal endometrium tissues and overexpression of miR-139-5p in endometrial cancer cells markedly reduced cell viability and migration, and suppressed HOXA10 expression. Our findings suggest that miR-139-5p may act as a tumor suppressor in endometrial cancer and miR-139-5p may be a potential therapeutic agent for endometrial cancer.

## Additional file

**Additional file 1.** The primers used for real-time PCR. The 3’-UTR of HOXA10 cDNA which contains a putative target region for miR-139-5p (bold stands for the putative target site for miR-139-5p).

## Abbreviations

EC: endometrial cancer
miRNA: microRNA
EMT: epithelial–mesenchymal transition
ESCC: esophageal squamous cell carcinoma
PCR: the real-time polymerase chain reaction
3ʹ-UTRs: 3ʹ-untranslated regions
