# Supplementary material for: Tumor-suppressor role of miR-139-5p in endometrial cancer
Source: Cancer Cell Int. 2018 Apr 2;18:51. doi: 10.1186/s12935-018-0545-8 (PMC5879796; doi:10.1186/s12935-018-0545-8)
Supplement: Supplementary file 1 — Additional file 1. The primers used for real-time PCR. The 3’-UTR of HOXA10 cDNA which contains a putative target region for miR-139-5p (bold stands for the putative target site for miR-139-5p). [file 12935_2018_545_MOESM1_ESM.docx]

**Additional file 1:** The primers used for real-time PCR. The 3′-UTR of HOXA10 cDNA which contains a putative target region for miR-139-5p (bold stands for the putative target site for miR-139-5p).

| Gene | Primer | Primer sequence |
| --- | --- | --- |
| U6 | Forward | CTCGCTTCGGCAGCACA |
|  | Reverse | AACGCTTCACGAATTTGCGT |
| Hsa-miR-139-5p | Forward | TCTACAGTGCACGTGTCTCCAGT |
|  | Reverse | TGGAGACACGTGCACTGTAGATT |

Dual-luciferase reporter assay

The sequence of 3'UTR of human HOXA10 cDNA containing the putative target site for the miR-139-5p; bold stands for the putative target site for miR-139-5p:

AGATATCCCAGCTTTAAAAAGAAAAAAAAAGAATTACCAAGAGAAGGGGACTTCTCTTCCAGTTTCTGTAAGGTCTTACATTGCCTGACTAAAATGTTTCATTTACCTCTAAATTTCCATATCCTTCTGG**CTGTAGA**TAAATAATGTAGTTTTGTTTATGCATTTGGAATTAGTGGATTTTTTTGTCATTAAAATTGTTACCACTGGTAACATGTGACAAGCACACCACAATTCTCCCTATCTTGTGAAGTTGTTTTTTTAAATCGCCTTGA
